# Tenth year reenrollment randomized trial investigating the effects of childhood probiotics and calcium supplementation on height and weight at adolescence

**DOI:** 10.1038/s41598-021-88819-y

**Published:** 2021-06-04

**Authors:** Evania Astella Setiawan, Davrina Rianda, Muzal Kadim, Meilianawati, Fenny Susanto, Frans J. Kok, Anuraj H. Shankar, Rina Agustina

**Affiliations:** 1 Department of Nutrition, Faculty of Medicine, Universitas Indonesia, Dr. Cipto Mangunkusumo General Hospital, Jl. Salemba Raya No.6, Jakarta, 10430 Indonesia; 2 Human Nutrition Research Center, Indonesian Medical Education and Research Institute (HNRC-IMERI), Faculty of Medicine, Universitas Indonesia, Jakarta, Indonesia; 3 Department of Pediatric, Faculty of Medicine, Universitas Indonesia, Dr. Cipto Mangunkusumo General Hospital, Jakarta, Indonesia; 4 Division of Human Nutrition and Health, Wageningen University, Wageningen, The Netherlands; 5 Centre for Tropical Medicine and Global Health, Nuffield Department of Medicine, University of Oxford, Oxford, United Kingdom; 6 Eijkman-Oxford Clinical Research Unit, Eijkman Institute for Molecular Biology, Jakarta, Indonesia

**Keywords:** Microbiology, Gastroenterology, Medical research

## Abstract

Microbiota and its modification with specific probiotics in early life could provide long term health benefits. Probiotics and calcium strengthen intestinal integrity and may support linear growth. This study investigated the long-term effects of childhood probiotics and calcium supplementation on growth in adolescence. We re-enrolled 238 adolescents aged 11–18 years from 494 children 10-years after 6-months of supplementation with either low-lactose milk fortified with low levels of calcium (LC, ∼50 mg/day, n = 53/124), with regular levels of calcium (RC, ∼440 mg/day, n = 70/126), or with regular calcium + 5 x 10^8^ CFU/day *Lactobacillus reuteri DSM 17938* (Reuteri, n = 55/124), or regular calcium + 5 x 10^8^ CFU/day *L. casei CRL 431* (Casei, n = 60/120). Changes in height-for-age z-score (HAZ) and body mass index-for-age z-score (BMIZ) were determined from the end of intervention to re-enrollment. General linear models were used to assess the effects on HAZ and BMIZ of group, gender, living area, maternal education, family income, physical activity, diet quality, nutritional status, and gut integrity as determined by urinary lactulose/mannitol ratio (L:M). Adolescent mean age was 15.3 years, mean HAZ was − 1.11, mean BMIZ was − 0.2 and median L:M (n = 155) was 0.23. Changes in HAZ and BMIZ were not significantly different between Casei, Reuteri, LC compared to RC. However, a significant decrease in BMIZ was observed among female adolescents in the Casei compared to RC group (− 0.5 SD, 95% CI − 0.8 to − 0.003, p = 0.048). Childhood probiotic and calcium supplementation may therefore selectively affect female adolescents.

**Clinical trial registration**: This follow-up study has been registered at www.clinicaltrials.gov, Registry name: Rina Agustina, Registration number: NCT04046289, First Registration Date 06/08/19. web link: https://www.clinicaltrials.gov/ct2/show/NCT04046289.

## Introduction

Several countries in Southeast Asia, including Indonesia, face the double burden of malnutrition^1^. According to a recent report by United Nations Children's Fund (UNICEF), Association of Southeast Asian Nations (ASEAN), and World Health Organization (WHO), 12% of children in Indonesia suffer from wasting and a further 12% are overweight, and both are public health challenges^2^. National nutrition policies to combat stunting, anemia, wasting and overweight in children and adolescents have been implemented, but have not yet shown impact^3^.

Malnutrition and infection have mutually synergistic adverse effects which could inhibit growth. Enteropathy is common in low resource environments^4^ and is often associated with an elevated lactulose mannitol ratio (L:M) indicating poor gut integrity, which in turn is correlated with gut dysfunction and growth failure^5^. Intestinal microbiota composition, and its modification with specific probiotics in early life, could provide long term health benefits^6^. Probiotics and calcium are sometimes used as food additives to strengthen intestinal integrity and may support child growth^7,8^. Probiotics may also improve the absorption of micronutrients such as calcium which are essential to support growth^9,10^, and potentially reduce stunting and malnutrition^11–13^. Studies assessing the long-term effects of probiotics supplementation during the perinatal period and early life stages have been carried out, and impact on growth has been inconclusive. This study was a 10-year re-enrollment follow-up of a previous randomized double blind clinical trial of probiotics and calcium supplementation for 6 months in children 1–6 years of age by Agustina et al.^7^. The intervention showed significant positive effects on weight gain, linear growth velocity, and reduction in stunting prevalence by 10% in the Reuteri (*Lactobacillus reuteri* DSM 17938) group. This re-enrollment study aimed to identify long-term effects of probiotics and calcium supplementation in childhood on growth during adolescence.

## Methods

### Study population and design

**Figure 1:**
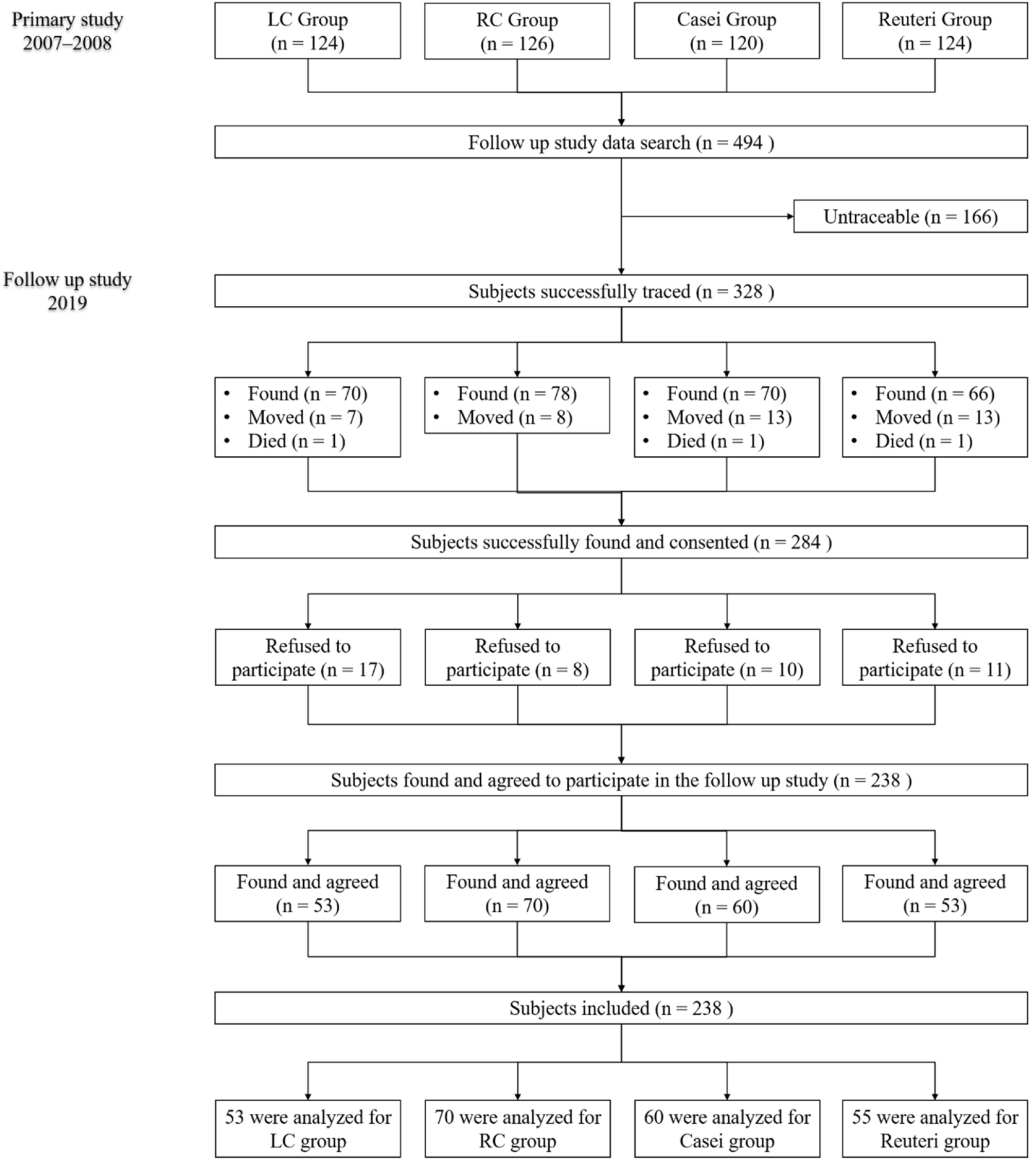
Flow diagram of follow-up study. Modified from Pediatrics. 2012;129:e1155–e1164.

This was an observational 10-year re-enrollment follow-up study examining the long-term effects of probiotics and calcium supplementation in children aged 1–6 years^7^. This study was conducted from January–May 2019 in selected flooding and non-flooding areas of East Jakarta, Indonesia. The previous study was a 6-month randomized, double-blind, placebo-controlled trial wherein healthy children were randomly assigned to receive low-lactose milk fortified with a low levels of calcium (LC, ∼50 mg/day, n = 124), or with regular levels of calcium (RC, ∼440 mg/day, n = 126), regular calcium with 5 × 10^8^ CFU/day *L. casei CRL 431* (Casei; n = 120), or regular calcium with 5 × 10^8^ CFU/day *L. reuteri DSM 17938* (Reuteri; n = 124). All participants from the previous study^7^ were eligible to re-enroll in the current study (Fig. 1. Consort Flow Diagram). First, we compiled sociodemographic information of 494 children from the previous intervention study^7^. We then contacted the local government officers and cadres (women community health volunteers), trained the field enumerators and traced all 494 children. All children who were successfully tracked were approached sequentially to participate in the re-enrollment study. Enumerators and participants were blinded from the beginning of the study until the end of the analysis, including all authors other than the original principal investigator (RA). Participants diagnosed with diabetes mellitus or being pregnant were excluded. Subjects who were unhealthy or consuming antibiotics had their re-enrollment postponed to one week after remission and completion of treatment. The outcomes for this study were growth measured by changes in HAZ and BMIZ from the end of the previous trial to assessment during adolescence at re-enrollment.

### Data collection

For re-enrollees, we collected data on socio-demographic and lifestyle characteristics such as age, gender, living area, maternal education, family income, physical activity questionnaire score for children (PAQ-C), and dietary quality index for adolescents score (DQI-A). Gut integrity was assessed as described below, and history of stunting and overweight/obese were assessed from the beginning of the intervention.

Trained enumerators were blinded to the allocation group and performed anthropometric measurements. Lightly clothed participants were weighed twice without shoes by using an electronic scale (SECA) with a precision of 0.1 kg. The height of subjects was measured twice by trained enumerators using a wooden Shorr board with a precision of 0.1 cm. Each subject was interviewed with the demographic and health characteristics questionnaire including PAQ-C. Usual dietary intake was assessed twice by 24-h recall which was used to calculate the DQI-A score (1 weekday and 1 weekend). Each subject was measured for height and weight, and then completed the lactulose/mannitol test while continuing with the interview to complete the questionnaires.

### Lactulose/mannitol ratio (L:M)

Out of the 4 intervention groups, only the Casei, Reuteri, and RC groups were analyzed for L:M, although urine collection was maintained for all group to ensure blinding. We performed the L:M assessment as a proxy for intestinal permeability related to probiotics administration, and therefore examined L:M only in the probiotics and control groups. Subjects were asked to do fasting for 10 h before the examination, and were gathered by the research team at a community meeting point in the district. Each subject first voided to empty the bladder, and then drank 100 mL of a solution containing 2 g mannitol (OTSU-MANITOL 20, mannitol 100 g per 500 mL) and 5 g lactulose (LACTULAX syrup, lactulose 3.335 g per 5 mL). Urine for each person was collected over the next 5 h in one jar with 1 mL chlorhexidine added to prevent bacterial degradation of the sugars. The time of ingestion of the sugar solution and the time of each urine collection was recorded. Upon completion of urine collection, the total volume was measured. A 60-mL aliquot was placed in a urine pot and transported at 4 °C to the laboratory and stored in a – 20 °C prior to analysis by High Performance Liquid Chromatography (HPLC). An increase in the urinary L:M ratio compared with the ratio administered reflects the degree of intestinal leakage^5^, and pathologically increased L:M was defined as greater than 0.1^14^.

### Ethical approval

The study protocol was approved by The Ethics Committee of the Faculty of Medicine, Universitas Indonesia (No: 1116/UN2.F1/ETIK/2018). All procedures were carried out in accordance with the International Conference on Harmonization-Good Clinical Practice (ICH-GCP). Separate approvals and authorization were also obtained from the head of the district and its public health office. Informed consent was obtained from all subjects and from a parent and/or legal guardian. The re-enrollment follow-up study is registered at www.clinicaltrials.gov (NCT04046289).

### Statistical analysis

Mean differences of changes in HAZ and BMIZ between groups were measured using Student’s t-test to identify differences in normally distributed variables between groups (Casei vs RC; Reuteri vs RC; and LC vs RC). For data that was not normally distributed, e.g. L:M, we used Mann-Whitney non-parametric tests. Generalized linear modelling was used to analyze several covariates such age, gender, living area, maternal education, family income, PAQ-C score, DQI-A score, stunting history, overweight/obese history, and L:M. All analyses were performed using Statistical Package for Social Sciences (SPSS) version 20.0. For all analyses, *p* < 0.05 was considered significant.

## Results

A total of 238 (Casei, 60 of 120; Reuteri 55 of 124; LC 53 of 124; RC, 70 of 126) out of 494 adolescents were re-enrolled. All groups were comparable with respect to socio-demographic and lifestyle characteristics, and nutritional status (Table 1). This follow-up study succeeded in re-enrolling almost 50% of subjects from January–May 2019 from the previous study that was completed nearly 10 years prior. Those re-enrolled were representative of the overall study population, and baseline characteristics for those found and not found were similar.

**Table 1:** Characteristics of adolescents according to assigned treatment.

	Casei n = 120		Reuteri n = 124		RC n = 124		LC n = 126	
	Found (n = 60)	Unfound (n = 60)	Found (n = 55)	Unfound (n = 69)	Found (n = 70)	Unfound (n = 54)	Found (n = 53)	Unfound (n = 73)
**Baseline characteristics of intervention study**								
Female, n (%)	27 (45)	27 (45)	35 (63.6)	21 (30.4)	36 (51.4)	22 (39.3)	28 (52.8)	29 (40.8)
Living in flooding area, n (%)	40 (66.7)	38 (63.3)	39 (70.9)	43 (62.3)	43 (61.4)	39 (69.6)	36 (67.9)	45 (63.4)
Low maternal education (> 9 years), n (%)	21 (35)	20 (33.9)	22 (40.7)	20 (29.4)	19 (27.1)	10 (18.2)	19 (35.8)	24 (33.8)
Stunting history^a^ n (%)	15 (25)	19 (31.7)	13 (23.6)	24 (34.8)	24 (34.3)	20 (37)	16 (30.2)	22 (30.1)
**Subject’s characteristics of follow-up study**								
Age at follow-up study (year)	15.4 ± 1.3^d^	–	15.3 ± 1.2^e^	–	15.3 ± 1.2	–	15.3 ± 1.3^f^	–
Sufficient family income^b^, n (%)	13 (21.7)^d^	–	10 (18.2)^e^	–	8 (11.4)	–	6 (11.3)^f^	–
PAQ-C Score	2.13 ± 0.5^d^	–	2.05 ± 0.5^e^	–	2.11 ± 0.5	–	2.18 ± 0.6^f^	–
DQI-A Score	34.9 ± 7.9^d^	–	34.7 ± 8.9^e^	–	32.0 ± 9.6	–	32.5 ± 8.8^f^	–
L:M^c^	0.23 (0.02–2.25)^d^ (n = 52)	–	0.26 (0.02–2.07)^g^ (n = 50)	–	0.23 (0.02–0.61) (n = 53)	–	N/A	–

Values are mean ± SD, or median (min–max), or n (%).

Unfound defined as subjects who moved, died, loss to follow-up and refused to participate in the follow-up study.

*DQI-A* diet quality index-adolescent, *L:M* lactulose mannitol ratio, *PAQ-C* physical activity questionnaire-children.

^a^Defined as a height-for-age Z-score < − 2 SDs before the intervention.

^b^Defined as family monthly income meeting the regional minimum wage.

^c^Tested and analyzed in 155 subjects.

^d^Casei compared to RC, analysis between groups, two tailed independent t-test analysis; P>0.05

^e^ Reuteri compared to RC, analysis between groups, two tailed independent t-test analysis; P>0.05

^f^ LC compared to RC, analysis between groups, two tailed independent t-test analysis; P>0.05

^g^Significantly different, Reuteri versus RC (*p* < 0.05); Mann-Whitney analysis.

### Dietary intake

The DQI-A scores were poor, and ranged from 32 to 35% which is half of the recommended normal value of 60%. The score was calculated from the average of 3 scores; the quality of food selection score (32%), dietary diversity score (58.4%), and dietary equilibrium score (11.4%), all of which were below recommended cutoffs.

### Growth status

No significant differences were observed overall for changes in height, weight, HAZ, and BMIZ between Casei, Reuteri, and LC groups compared to control (Table 2). However, analyses revealed that other risk factors contributed to the outcomes (Table 3). Age, stunting history, and overweight/obese history variables contributed to the change in HAZ, whereas age, gender, stunting history, and overweight/obese history contributed to the change in BMIZ. In the original study, stratified analyses were carried out based on age, gender, and location. We therefore stratified on those variables in this study (Table 4), which revealed a significant effect of *L. casei* in reducing the change in BMIZ for females (− 0.5 SD, 95% CI − 0.8 to − 0.003, *P* = 0.048).

**Table 2:** Long term effects on growth in adolescents who consumed low lactose milk in their childhood.

Outcome measures	Groups				Adjusted difference (95% CI)					
	Casei ^†^ n = 60	Reuteri ^†^ n = 55	RC ^†^ n = 70	LC ^†^ n = 53	Casei vs. RC^a^ n = 130	p-value	Reuteri vs. RC^a^ n = 125	p-value	LC vs. RC^a^ n = 123	p-value
**Changes in 10 years**^b^										
Height	52.9 ± 9.3	51.9 ± 7.8	53.1 ± 9.3	54.1 ± 8.5	1.5 (− 2.1–5.2)	0.41	1.9 (− 2.3–6.2)	0.36	1.6 (− 1.9–5.1)	0.38
Weight	35.2 ± 9.4	35.8 ± 10.2	35.0 ± 9.9	33.4 ± 7.6	0.1 (− 3.1–3.4)	0.93	1.1 (− 2.5–4.6)	0.56	1.5 (− 1.6–4.6)	0.35
HAZ	0.1 ± 1.1	0.1 ± 0.7	0.1 ± 0.9	0.2 ± 1.0	− 0.1 (− 0.4–0.2)	0.46	− 0.001 (− 0.3–0.3)	0.99	− 0.1 (− 0.4–0.2)	0.61
BMIZ	− 0.1 ± 1.0	0.3 ± 1.0	0.3 ± 1.2	0.1 ± 1.0	− 0.3 (− 0.7–0.04)	0.08	− 0.01 (− 0.4–0.4)	0.95	0.1 (− 0.2–0.5)	0.44

*BMIZ* body mass index-for-age z-score, *CI* confidence interval, *HAZ* height-for-age z-score.

^†^Mean ± SD (all such values).

^a^General linear model, adjusted for age, gender, and living area.

^b^Changes between the end of intervention and after 10-years follow up measurements.

**Table 3:** Multiple regression model of each risk factor for outcome changes in HAZ and BMIZ.

	HAZ changes^a^ (n = 238)	BMIZ changes^a^ (n = 238)
**Sosiodemographic and lifestyle risk factors**		
Age	− 0.16 (− 0.26 to − 0.05)*	0.11 (0.02 to 0.20)*
Gender	− 0.11 (− 0.39 to 0.18)	0.39 (0.12 to 0.65)*
Living in flooding area	− 0.11 (− 0.37 to 0.16)	0.03 (− 0.22 to 0.28)
Low maternal education (< 9 years)	− 0.07 (− 0.35 to 0.20)	0.16 (− 0.10 to 0.41)
Sufficient family income	− 0.35 (− 0.71 to 0.01)	− 0.20 (− 0.54 to 0.13)
PAQ-C Score	0.01 (− 0.26 to 0.28)	− 0.09 (− 0.34 to 0.16)
DQI-A Score	0.01 (− 0.01 to 0.02)	− 0.01 (− 0.02 to 0.01)
**Gut integrity and nutritional status**		
L:M^b^	0.17 (− 0.26 to 0.59)	− 0.04 (− 0.60 to 0.52)
Stunting history	0.61 (0.38 to 0.85)*	− 0.32 (− 0.62 to − 0.01)*
Overweight/obese	− 0.93 (− 1.40 to − 0.47)*	− 0.79 (− 1.40 to − 0.18)*

*BMIZ* body mass index-for-age z-score, *DQI-A* diet quality index-adolescent, *HAZ* height-for-age z-score, *L:M* lactulose/mannitol ratio, *PAQ-C* physical activity questionnaire-children.

^a^Values are standardized effect sizes.

^b^Tested and analyzed in 155 subjects.

*Generalized linear model with p-value < 0.05.

**Table 4:** Outcomes measurement in adolescents stratified with age, gender, location.

	Adjusted difference (95% CI) for HAZ changes			Adjusted difference (95% CI) for BMIZ changes		
	Casei vs. RC n = 130	Reuteri vs. RC n = 125	LC vs. RC n = 123	Casei vs. RC n = 130	Reuteri vs. RC n = 125	LC vs. RC n = 123
**Age (years)**						
≤ 15	− 0.1 (− 0.6 to 0.4)	− 0.1 (− 0.6 to 0.4)	− 0.1 (− 0.6 to 0.4)	− 0.3 (− 0.9 to 0.2)	− 0.1 (− 0.6 to 0.5)	− 0.03 (− 0.5 to 0.5)
> 15	− 0.2 (− 0.5 to 0.1)	0.1 (− 0.3 to 0.4)	0.2 (− 0.1 to 0.6)	− 0.3 (− 0.8 to 0.1)	0.1 (− 0.4 to 0.5)	− 0.3 (− 0.8 to 0.2)
**Gender**						
Male	− 0.2 (− 0.7 to 0.4)	− 0.1 (− 0.7 to 0.4)	− 0.01 (− 0.5 to 0.5)	− 0.2 (− 0.7 to 0.3)	0.3 (− 0.3 to 0.9)	0.1 (− 0.4 to 0.6)
Female	− 0.1 (− 0.4 to 0.3)	0.1 (− 0.2 to 0.5)	0.2 (− 0.2 to 0.6)	− 0.5 (− 0.9 to − 0.003)*	− 0.2 (− 0.7 to 0.2)	− 0.4 (− 0.9 to 0.1)
**Location**						
Flooding area	− 0.3 (− 0.7 to 0.1)	− 0.1 (− 0.5 to 0.3)	0.1 (− 0.4 to 0.5)	− 0.3 (− 0.7 to 0.1)	0.1 (− 0.3 to 0.5)	− 0.1 (− 0.5 to 0.3)
Non-flooding area	0.2 (− 0.2 to 0.7)	0.1 (− 0.4 to 0.6)	0.1 (− 0.4 to 0.6)	− 0.4 (− 1 to 0.3)	− 0.4 (− 1.1 to 0.3)	− 0.3 (− 0.9 to 0.4)

*BMIZ* body mass index-for-age z-score, *CI* confidence interval, *HAZ* height-for-age z-score, *PAQ-C* physical activity questionnaire-children.

Generalized linear model was performed in all such values; *p < 0.05.

### Gut integrity

 The median L:M value for 155 subjects tested was 0.23 (0.02-2.25). Most L:M (89.2%) were categorized as having poor gut integrity indicating the high prevalence of Environmental Enteric Dysfunction (EED) in this population. Significant differences were found in L:M between groups, wherein subjects in the Reuteri group tended to have poorer gut integrity. We note that no side effects were reported after the administration of the lactulose mannitol solution

## Discussion

This ten-year re-enrollment randomized trial investigating the long-term effects of childhood probiotics and calcium supplementation on growth among adolescents demonstrated there were no significant differences in overall outcomes between groups before and after adjustment for confounding factors. However, there was significant reduction in BMIZ for female subjects supplemented with *L. casei CRL 431*.

Few follow-up studies have been carried out to investigate long-term effects of single-strain probiotics on growth. These studies differed in subjects, probiotic strain, dose, and duration of intervention, and showed varying results. A follow up study of 5-months oral *L. fermentum CECT 5716* supplementation in healthy 1-month-old infants assessed the linear growth outcomes at 6 months, 1 year, 2 years, and 3 years after supplementation. They reported taller subjects at 6 months, 1 year, and 2 years, wherein the difference in length between groups gradually reduced over time, and with no significant difference at 3 years after supplementation^15^. A review by Derrien et al.^16^ concluded that the activity of *Lactobacillus* in the gastrointestinal tract and its effect on composition and metabolism of the microbiome is transient and diminishes over time after supplementation is stopped.

Another follow up study of 1-year of *L. rhamnosus GG* supplementation of healthy infants showed no significant differences in height and weight between the probiotic and control group after 3 and 5 years^17,18^. In a study in families with a history of allergy, intervention with *L. reuteri ATCC 55730* was initiated during pregnancy and continued with child supplementation postnatally until 12 months of age, and follow-up at 7 years of age showed no significant difference in height or weight between groups^19^. Overall, follow-up studies of probiotic supplementation on growth have been inconclusive, but suggest the type of strain and age at supplementation may be important factors.

In our study, we note that a non-significant increase was observed in HAZ in the probiotic group compared to the control group. The LC group similarly showed a non-significant increase in HAZ compared to the RC group. Probiotic may interact with calcium, zinc, and iron to enhance growth. With regards to calcium, there are reports that calcium supplementation had no significant effect on the linear growth of children nor any lasting effect on bone mineral or bone size in the latter life^4,20–22^. We note that the absorption and balance of zinc and iron can also be affected by probiotics, calcium and other micronutrients ^23–27^; although, such interactions were not observed in this study. Further research is needed to investigate the effects of probiotic and calcium supplementation on iron and zinc absorption in adolescents, preferably using more advanced methods such as stable isotope techniques.

This study showed a significant decrease in BMIZ in females for the Casei group compared to control (Table 4). This is supported by another study in obese children given *L. casei strain Shirota* (LcS)-containing beverages. A significant decline in body weight was observed 6 months after ingestion of the LcS beverage compared to control^28^. Probiotics have an important role in maintaining healthy intestinal microbiota which can provide an anti-obesity effect^29^. The latest systematic review and meta-analysis states that only certain strains of probiotics may regulate body weight^30^. Consumption of fermented milk containing LcS improves the intestinal environment, such as increasing the concentration of acetic acid and/or decreasing the intestinal pH which ultimately increases the number of *Bifidobacteria* colonies, which are important in preventing the proliferation of destructive bacteria in the intestine. In addition to the bactericidal effect, the high colony number of *Bifidobacteria* after consuming LcS promotes fermentation in the intestine, decreases food intake and fat mass, and improves insulin secretion which ultimately helps to reduce weight gain^28^. Oral administration of probiotic facilitates thermogenic and lipolytic effects through stimulating the sympathetic nervous system, which leads to weight reduction^31^. On the other hand, the LC group in our study tended toward BMIZ reduction in female adolescents compared to the RC. Calcium is well known for its effect in reducing body mass index (BMI) or fat mass as a protective effect in relation to childhood obesity^32,33^. Although much of the published clinical data supports a negative association between calcium intake and body fat mass, others have found no evidence for an effect of calcium intake on body weight or fat mass ^34,35^. Gunther et al.^34^ demonstrated that increase in calcium intake was not associated with weight loss and fat mass reduction in healthy young women after 1 year. At the mechanistic level, calcium may regulate adipocyte metabolism through suppressing parathyroid hormone, and through 1,25-hydroxy vitamin-D which may inhibit lipogenesis and stimulate lipolysis^36^.

In this study, 89.2% of subjects had L:M above the normal ratio, with a median L:M of 0.23 (0.02–2.25). L:M is the most widely implemented surrogate marker of EED, and reflects important components of the gastrointestinal barrier and absorptive function^37,38^. Our results show the high prevalence of tropical enteropathy in this population. This is in line with an observational study conducted in Indonesia by Pusponegoro et al.^39^ which assessed intestinal permeability in 62 healthy children aged 2–10 years, and showed a median L:M of 3.63 (0–100), indicating a high prevalence of EED that could be a confounder in probiotic interventions. EED is characterized by chronic villous atrophy, crypt hyperplasia, and inflammatory cell infiltration which causes increased intestinal permeability and bacterial translocation. Environmental factors and poor sanitation play an important role for this phenomenon. Bacterial translocation induces recurrent infections, decreased nutrient absorption capacity and changes in the immune system that ultimately lead to nutritional deficiencies in children that affect growth^40^. In our regression model, the L:M did not influence the effect of probiotic and calcium supplementation on growth, while the history of nutritional status did influence growth. Gut integrity is closely related to the microbiome in which the microbial diversity increases and converges toward an adult-like microbiota by the end of the first 3–5 years of life^41^. Once established, the composition of the gut microbiota is relatively stable throughout adult life, hence it might not be easily changed by intervention. Further studies are needed with L:M as the primary outcome to assess the effects of probiotics on gut integrity.

In the original study^7^, stratified randomization was carried out to assure comparable baseline characteristics among groups. In addition, the intervention was carried out over 6 months, sufficient to observe any growth effects. Assessment of growth in the original and current studies was by trained professional staff who were blinded. Despite this high quality implementation, a limitation of the current study was the extended 10-year interval after the original study, wherein many intervening factors could affect the outcomes, and also bias recall toward a null finding. We observed 50% lost-to-follow-up in each group, and took this into account by analyzing the subject characteristics amongst those found and unfound, which showed no differences. Most of those lost-to-follow-up moved outside Jakarta and refused to participate in the re-enrollment study. Reasons for refusal were because of the long period of time required for data collection, and conflict with school activities on weekends and weekdays. Two subjects were reported as deceased, one due to illness and another to a traffic accident. There were no reported adverse events from the previous study. On the contrary, nearly all mothers declared their perception of the beneficial effects on their children’s growth, irrespective of treatment group. We note we did not analyze the gut microbiome to assess possible long term changes in intestinal colonization by the probiotic organism and its association with long-term effects.

The routine use of probiotics may lead to improve public health outcomes, especially in developing countries, by supporting growth and preventing obesity in children and adolescents. Moreover, there is emergent research assessing how gender influences the effects of probiotics interventions in obesity. This research is expected to stimulate more clinical trials in the field of probiotics with application of reliable methods and standardized surrogate markers. Further studies are needed to assess the short and long term effects of probiotics on growth as a main outcome. The follow-up assessments should be conducted in shorter and repeated periods of time, thereby enhancing quality and completeness of data within that time period.

## Conclusion

There were no significant overall differences in changes of height, weight, HAZ, or BMIZ between groups before and after adjustment for confounders. A significant effect of BMIZ reduction was identified in female adolescents who were supplemented in childhood with probiotic *L. casei CRL 431* and regular calcium levels. This outcome may represent reduced risk for obesity later in life which warrants further exploration*.*

## Data Availability

The identified individual participant data will not be made available.

## Abbreviations

HAZ: Height-for-age Z-score
BMIZ: BMI-for-age Z-score
L:M: Lactulose/mannitol ratio
